# Symptoms in the general Norwegian adult population - prevalence and associated factors

**DOI:** 10.1186/s12889-020-09109-2

**Published:** 2020-06-23

**Authors:** Hilde Krogstad, Jon Håvard Loge, Kjersti S. Grotmol, Stein Kaasa, Cecilie E. Kiserud, Øyvind Salvesen, Marianne Jensen Hjermstad

**Affiliations:** 1 European Palliative Care Research Centre (PRC), Department of Clinical and Molecular Medicine, Faculty of Medicine and Health Sciences, NTNU, Norwegian University of Science and Technology, and St. Olavs hospital, Trondheim University Hospital, Trondheim, Norway; 2 Cancer Clinic, St. Olavs hospital, Trondheim University Hospital, Trondheim, Norway; 3 Regional Advisory Unit in Palliative Care, Department of Oncology, Oslo University Hospital, Oslo, Norway; 4 European Palliative Care Research Centre (PRC), Department of Oncology, Oslo University Hospital and Institute of Clinical Medicine, University of Oslo, Oslo, Norway; 5 Institute of Basic Medical Sciences, Faculty of Medicine, University of Oslo, Oslo, Norway; 6 National advisory unit for late effects after cancer treatment, Oslo University Hospital, and University of Oslo, Oslo, Norway; 7 Department of Public Health and Nursing, Faculty of Medicine and Health Sciences, NTNU, Norwegian University of Science and Technology, Trondheim, Norway

**Keywords:** Patient reported outcome measures, PROMS, MDASI, Reference values

## Abstract

**Background:**

Patients´ own perceptions and evaluations of symptoms, functioning and other health-related factors, i.e. Patient Reported Outcomes (PROs), are important elements for providing good patient care. Symptoms are subjective and best elicited by the patient orally or by using PRO measures (PROMs), be it on paper, or as electronic assessment tools. Reference values on frequently used PROMs facilitate the interpretation of scores for use in clinics and research settings, by comparing patient data with relevant samples from the general population. Study objectives were to (1) present reference values for the M.D. Anderson Symptom Inventory (MDASI) (2) examine the occurrence and intensity of symptoms assessed by the MDASI in a general Norwegian adult population sample, and (3) examine factors associated with higher symptom burden defined as the sum score of all symptoms, and factors associated with symptoms` interference on functions.

**Methods:**

In 2015, MDASI was sent by mail as part of a larger survey, to a representative sample of the general Norwegian adult population (*N* = 6165). Medical comorbidities were assessed by the Self-Administered Comorbidity Questionnaire. Depression was self-reported on the Patient Health Questionnaire 9 (PHQ-9). Linear multivariable regression analysis was used to examine for factors associated with MDASI sum score and factors associated with symptoms’ interference on functions.

**Results:**

The response rate was 36%. More women (54%) than men (46%) responded. Mean age was 55 years (SD 14). The most frequent symptoms were fatigue (59.7%), drowsiness (56.2%) and pain (56.1%). Fatigue, pain and disturbed sleep had the highest mean scores. The presence of one or more comorbidities, increasing PHQ-9 score and lower level of education were associated with higher MDASI sum score (*p* < 0.001). The MDASI sum score and the PHQ-9 score were positively associated with all interference items (*p* < 0.001) except for walking (*p* = 0.22).

**Conclusion:**

This study provides the first Norwegian reference values for MDASI. The presence of one or more comorbidities, higher level of depressive symptoms and lower level of education were significantly associated with higher MDASI sum score. These covariates must be controlled for when using the reference values.

## Background

Patient Reported Outcomes (PROs) are patients´ own perceptions and evaluations of symptoms, functioning and other health-related factors, and are important elements for providing good patient care [1]. A symptom is defined as any subjective evidence of a disease, health condition, or treatment-related effect that can be noticed and known only by the patient [1]. In contrast, a “sign” is any objective evidence of disease that can be identified by health care personnel by observations, examinations, biomarkers, imaging etc. or may be noticed and reported by the patient [1]. Symptoms may indicate the presence of a disease or a disorder but may also reflect normal variations in physical or psychological states as commonly experienced by most individuals. Symptoms are common in the general population [2–5]. A large Danish nationwide cohort study with 49, 706 respondents representative of the general population demonstrated that symptoms were common; about 9 out of 10 respondents reported at least one symptom within the preceding 4 weeks [2]. Other population studies have reported that 75 and 90% had experienced at least one symptom in the previous 2 weeks and 30 days respectively [3, 5]. Some symptoms have low positive predictive value for disease while others are stronger predictors [6]. As this may vary for different symptoms across patient populations, reference values from the general population provide important information about the predictive values of symptoms for disease. The prevalence of symptoms in the general population is found to be associated with factors such as chronic conditions, age, employment status, living situation and psychiatric disorders [3, 7]. The number of symptoms is also documented to have a linear relationship with functional status [4].

Patient-Reported Outcome Measures (PROMs) denote any standardized measure of a PRO, i.e. a questionnaire, of a patient’s health and quality of life (QoL) [8]. These questionnaires are intended for self-completion by patients, in the form of the traditional paper forms or more recently in electronic formats (e-PROMs) for use on different platforms, e.g. cell phones, computers, tablets etc. [9]. PROMs provide information that comes directly from the patient [8]. In clinical care, PROMs can be used alongside laboratory tests and imaging, if properly assessed and followed. Regular and systematic use of PROMs may improve communication between patients and health care providers [10] and be used to monitor treatment response and detect unrecognized problems or problems not reported spontaneously by the patient [11]. Beyond their clinical utility, PROMs are increasingly being used as outcomes in epidemiologic, health economic and clinical research [12]. PROMS are also central components of patient-centered care [13, 14]. Recent studies suggested that active use of PROMs during treatment for advanced cancer may even prolong survival [15–17].

Clinicians or researchers often request reference data to facilitate the interpretation of patient data or study results [18]. Reference values for PROMs facilitate the interpretation of PROMs scores both in clinics and research settings, by comparing patient data with relevant samples from the general population. Reference values may also be used to evaluate the relative symptom burden of a disease in a given diagnosis, when controlled after adjusting for relevant covariates [19]. Hence, a number of datasets with population-based reference data have been published and are frequently being used, e.g. the Patient-Reported Outcomes Measurement Information System [19], European Organisation for Research and Treatment of Cancer (EORTC) Core Quality of Life Questionnaire C30 [20, 21] and the Functional Assessment of Cancer Therapy-General [22]. Reference values make comparisons between samples possible, but this requires adjusting for known variables that affect the outcomes, e.g. age, sex, residence, education, comorbidities and other sociodemographic variables [20, 23]. As reference values are based on self-report, as are patient-reported outcomes, there is not and should not be, a golden standard for a given symptom score as is the case. In contrast to e.g. reference values for laboratory results, the principle of PROs as part of patient-centered care is to assess the patients’ own perception of symptoms and QoL. As such, reference data provide information about the distribution of self-reported QoL scores for given reference populations. These scores can be used as reference against which patient scores can be compared. If the average score in a patient group is significantly higher or lower than expected after controlling for known covariates, follow-up of potential disease or treatment side effects may be indicated [24]. The relevance of valid reference data is illustrated in follow-up studies among cancer survivors, which may go beyond 20 years post-treatment [25, 26]. During such a long period, common age-related health problems and life events may influence which symptoms the cancer survivors experience and how they perceive their QoL and level of functioning. By comparing with data from the general population one can ascertain if cancer survivors are at excess risk for specific symptoms and health problems compared to individuals with similar age, sex and other background variables.

The M.D. Anderson Symptom Inventory (MDASI) is a brief, reliable and valid tool for self-report of symptoms commonly experienced by patients with cancer and also assesses their impact on daily functioning [27]. The MDASI is frequently used in clinical cancer care [28, 29]. Importantly, all MDASI symptoms are prevalent in the general population and how self-reported severity of symptoms interfere daily living is an important issue in all populations. Reference values for the MDASI from the general adult population therefore allow for interpretation of scores from patient samples and for comparison across studies and between relevant populations samples. Up until now, there are no reference values for the MDASI from the Norwegian population, nor have we found this from other countries.

On this background, study objectives were to (1) present reference values for the M.D. Anderson Symptom inventory (MDASI), (2) examine the occurrence and intensity of symptoms assessed by the MDASI in a general Norwegian adult population sample, and (3) examine factors associated with higher symptom burden defined as the sum score of all symptoms, and factors associated with symptoms` interference on functions.

## Methods

### Data collection

In the spring 2015, 6165 subjects, aged 18–80 years, and representative of the general Norwegian adult population with respect to age, gender and place of residence, were randomly drawn by Bring Dialog [30]. They received a mailed questionnaire packet on paper containing the Short-Form Health Survey-36 (SF-36), version 1 [31, 32], the M.D. Anderson Symptom Inventory (MDASI) [27], the Fatigue Questionnaire (FQ) [33] and the Patient Health Questionnaire-9 (PHQ-9) [34, 35]. The questionnaire packet also included questions covering 13 comorbidities [36] and 14 questions related to socio-demographic variables, physical activity, general health and contact with health care providers. Socio-demographic variables (see below), comorbidities, the MDASI and the PHQ-9 were used in this study.

### Socio-demographic variables

Socio-demographic variables included year of birth, sex, and level of education. Education was divided into three groups referring to highest level of completed education: elementary and/or primary school; second level (high school); and third level (university college or university). Comorbidities were self-reported on a modified version of the Self-Administered Comorbidity Questionnaire (SCQ) [36]. The subjects were asked “do you have, or have you ever had, any of the following diseases/problems?”

### Instruments

#### The M.D. Anderson symptom inventory (MDASI)

The M. D. Anderson Symptom Inventory (MDASI) was developed by the Pain Research Group at M. D. Anderson Cancer Center at the University of Texas. Validation studies have shown that the MDASI is useful for symptom surveys, clinical trials, and patient follow-up care [28, 37, 38]. MDASI is designed for use in cancer populations [27], hence applies to patients with various cancer diagnoses and types of treatment. MDASI assesses the severity of 13 frequent symptoms experienced during the last 24 h (pain, fatigue, nausea, sleep disturbance, distress, shortness of breath, difficulty remembering, lack of appetite, drowsiness, dry mouth, sadness, vomiting, numbness/tingling) in patients with cancer. The response alternatives are 0–10 on numerical rating scales, with 0 meaning “not present” and 10 meaning “as bad as you can imagine”. In this study, a cut off ≥1 was chosen to denote any presence of a symptom. These 13 items not only account for the most frequently reported symptoms by cancer patients, but they are also common reasons for contact with the health care system in the general population [27, 39]. In addition, the MDASI includes another six questions on how much the symptoms interfere with general activity, mood, work, relations with other people, walking and enjoyment of life. The interference items are also measured on 0–10 scales, with 0 meaning “did not interfere,” and 10 meaning “interfered completely”. The first introductory sentence in the MDASI refers to people with cancer *“people with cancer frequently have symptoms that are caused by their disease or by their treatment”*. For the purpose of this survey, the sentence was changed to: “*many people often have symptoms due to injuries or disease*”. Thus, the word cancer was omitted from the questionnaire.

The translation of MDASI into Norwegian followed the multi-step, well-established 2009 procedures developed by the EORTC Quality of Life Group [40]. This includes two independent forward translations from English to Norwegian by native speakers of Norwegian language with good knowledge of English. A third person fluent in both languages merged the translations into a reconciled version, that was back-translated by two persons having a very good command of English. When comparing the original and the back-translated English versions, no translation problems became apparent. The Norwegian version of the MDASI was proof-read and pilot-tested by six persons who found the comprehensibility and clarity satisfactory according to the EORTC debriefing interviews (length, relevance, confusing, upsetting and intrusive items, unclear wording) [40]. Permission to translate and use the MDASI was obtained from MD Anderson, TX, USA.

#### The patient health Questionnaire-9 (PHQ-9)

PHQ-9 is a nine-item questionnaire designed to screen for depression [35]. The nine items correspond to the DSM-5 diagnostic criteria for major depressive disorder [41]. The response alternatives are the frequency to which these symptoms have been bothersome during the past 2 weeks, divided in four categories: 0 = *not at all*, 1 = *several days*, 2 = *more than half of the days* and 3 = *nearly every day*. “Major depression” is diagnosed if five or more of the symptoms have been present at least “more than half the days” in the past 2 weeks provided that one of these is item 1 (depressed mood) or item 2 (anhedonia). As a severity measure, the PHQ-9 score ranges from 0 to 27, since each item can be scored from 0 to 3. In the present study, the four somatic depression symptoms in the PHQ-9 are excluded to avoid overlap with MDASI (sleep-problems, fatigue, weight/appetite change and psychomotor retardation). The instrument will hereafter be referred to as the PHQ. Here, the score ranges from 0 to 15. We have previously shown that the agreement between the 9 - and 5 - item versions in detecting depression was excellent [42].

### Statistical analysis

The returned questionnaires that were blank, had no data on sex or missed more than half of the individual MDASI symptoms were excluded from analysis. Standard descriptive analyses were used with the baseline characteristics. Variables examined included age, gender and education. The number of age groups was limited to six: 18–29, 30–39, 40–49, 50–59, 60–69, and 70–80 years. The number of comorbidities were grouped as follows: Category 0 (no comorbidity), category 1 (1–2 comorbidities) and category 2 (≥ 3 comorbidities). Basic descriptive analyses were used for the number and intensity of MDASI symptoms. The total MDASI sum score for the 13 symptoms was calculated (possible range 0–130; the sum of scores for the 13 individual symptoms).

Associations between the MDASI sum score as the dependent variable, and age, sex, education, comorbidity and depression as independent variables were analyzed using linear multivariable regression. Univariable linear regression was used to examine for factors associated with MDASI sum score. Variables from the univariable analyses with a *p*-value ≤0.10 were included in the multivariable regression model, which also included sex and age regardless of the significance in the univariable analyses. The six MDASI interference items were used as dependent variables in separate analyses. The corresponding effect sizes are reported as unstandardized coefficients and 95% confidence interval (CI). A *p*-value of < 0.05 was used to denote statistical significance.

The statistical software applied was IBM SPSS Statistics for Windows, version 25.0, (IBM Corporation, Armonk, NY, USA).

### Ethical considerations

The study was performed according to the rules of the Helsinki declaration. All respondents received written information about the study. Return of the questionnaires was taken to indicate written, informed consent. The Regional Committee for Medical and Health Research Ethics (REC) South East Norway approved the survey (2014/1172).

## Results

The overall response rate was 36%. Of the 2130 returned questionnaires, 23 were blank, 21 had no data on sex, and 65 had responded to less than half of the individual MDASI symptoms. All these respondents were omitted, giving a sample of 2021. Missing values of the MDASI ranged from 0.1% (*n* = 3, numbness) to 1.4% (*n* = 28, fatigue).

More women (54%) than men (46%) responded. As shown in a previous publication from the same material [32], the response rate for both men and women was 5% in the youngest age group (≤ 29 years) which was significantly lower compared to the other groups (*p* < 0.001). Mean age of the study sample was 55 years (SD 14) (Table 1). Forty-six% of the respondents had university college or university education.

**Table 1:** Socio-demographic characteristics, and mean MDASI sum score

Variables	Population (*N* = 2021)	Mean MDASI sum score (SD)^a^
Age		
Mean (±SD)	55 (14)	
Min.-Max.	18–79	
Age groups, N (%)		
≤ 29 years	101 (5.0)	18.78 (20.24)
30–39 years	197 (9.7)	15.76 (18.89)
40–49 years	390 (19.3)	14.68 (18.15)
50–59 years	467 (23.1)	15.46 (17.88)
60–69 years	499 (24.7)	15.13 (18.63)
≥ 70 years	367 (18.2)	15.84 (17.91)
Gender, N (%)		
Women	1101 (54)	16.71 (18.83)
Men	920 (46)	14.03 (17.65)
Education, N (%), Missing 10 (0.5)		
Elementary and/or primary school	344 (17.1)	18.63 (20.55)
Second level (high school)	751 (37.3)	16.98 (19.57)
Third level (university college or university)	916 (45.5)	12.98 (15.95)
Number of comorbidities, N (%)		
0	856 (42)	
1–2	912 (45)	
≥ 3	253 (13)	

^a^Min-max 0–130

Table 2 shows the frequency of comorbidities. Forty-two% reported no comorbidities, 45% reported one or two while 13% reported three comorbidities or more. The most frequent were hypertension, arthrosis and depression. Arthrosis and depression were more common in women (23.6 and 15.3% vs. 12.5 and 9.3%), while there was no difference regarding hypertension between men and women. Depression was more common among women in the youngest age group (23.1%) compared to women ≥70 years (15.3%).

**Table 2:** Comorbidities ^a^, overall and by sex

Comorbidity	All N (%)	Women N (%) *N* = 1101	Men N (%) *N* = 920
Heart disease	135 (6.7)	34 (3.1)	101 (11.0)
Hypertension	482 (23.8)	262 (23.8)	220 (23.9)
Chronic lung disease	205 (10.1)	116 (10.5)	89 (9.7)
Diabetes	113 (5.6)	44 (4.0)	69 (7.5)
Kidney disease	40 (2.0)	17 (1.5)	23 (2.5)
Liver disease	23 (1.1)	9 (0.8)	14 (1.5)
Stomach/Bowel disease	123 (6.1)	62 (5.6)	61 (6.6)
Rheumatic disease	145 (7.2)	100 (9.1)	45 (4.9)
Arthrosis	375 (18.6)	260 (23.6)	115 (12.5)
Epilepsy	22 (1.1)	16 (1.5)	6 (0.7)
Stroke	60 (3.0)	27 (2.5)	33 (3.6)
Depression	259 (12.8)	169 (15.3)	90 (9.8)
Other psychiatric conditions	155 (7.7)	99 (9.0)	56(6.1)

^a^The Self-Administered Comorbidity Questionnaire [36]

**Fig. 1:**
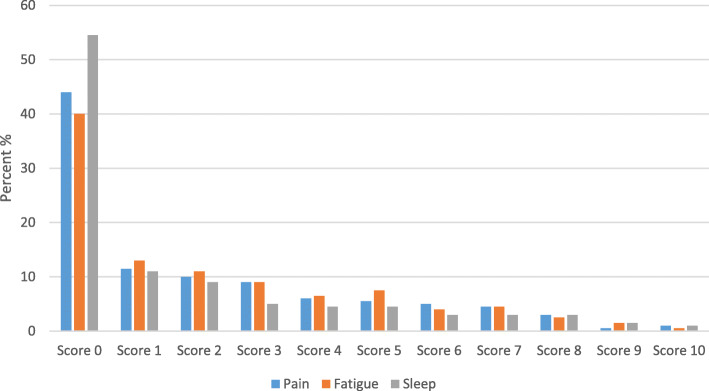
Distribution of scores 0–10 on pain, fatigue, sleep

The most frequent symptoms were fatigue (59.7%), drowsiness (56.2%) and pain (56.1%). When using a cut off ≥3, the prevalence was 34.8% for fatigue, 34.2% for pain and 26.7% for drowsiness (Table 3). The mean scores for the 13 symptoms by age and sex are presented in Table 4. Fatigue, pain and disturbed sleep had the highest mean scores overall (Fig. 1). Fatigue had the highest mean score; 2.39 in women and 1.90 in men. The mean scores for fatigue were highest in the youngest age group (< 30 years), with higher score for women (3.45) than in men (2.36). Overall, the mean scores for pain were 2.24 in women and 1.94 in men, and the mean scores for disturbed sleep were 1.93 in women and 1.42 in men.

**Table 3:** Frequency of symptoms (MDASI score), N (%)

Symptom	MDASI score ≥ 1	MDASI score ≥ 3
Pain	1125 (56.1)	692 (34.5)
Fatigue (tiredness)	1190 (59.7)	704 (35.3)
Nausea	305 (15.3)	134 (1.3)
Disturbed sleep	913 (45.5)	507 (25.3)
Being distressed	913 (45.5)	433 (21.6)
Shortness of breath	600 (30.0)	289 (14.4)
Remembering	699 (34.9)	276 (13.8)
Lack of appetite	357 (17.8)	148 (7.4)
Drowsy	1127 (56.2)	540 (26.9)
Dry mouth	578 (28.8)	285 (14.2)
Sad	789 (39.2)	374 (18.6)
Vomiting	164 (8.1)	69 (3.4)
Numbness or tingling	503 (24.9)	265 (13.1)

**Table 4:** Mean MDASI scores (SD)^a^ by sex and age groups, *N* = 2021

Symptoms	Age groups												Total	
	18–29 years		30–39 years		40–49 years		50–59 years		60–69 years		70–80 years			
	W	M	W	M	W	M	W	M	W	M	W	M	W	M
	(*n* = 64–65)	(*n* = 36)	(*n* = 115–116)	(*n* = 80–81)	(*n* = 222–227)	(*n* = 162–163)	(*n* = 251–257)	(*n* = 207–210)	(*n* = 242–248)	(*n* = 248–251)	(*n* = 182–188)	(*n* = 174–179)	(*n* = 1084–1101)	(*n* = 914–920)
Core items														
Pain	1.69 (2.44)	1.17 (2.12)	1.69 (2.49)	1.64 (2.35)	2.22 (2.71)	1.90 (2.34)	2.39 (2.65)	1.97 (2.58)	2.53 (2.78)	2.04 (2.46)	2.21 (2.58)	2.10 (2.51)	2.24 (2.66)	1.94 (2.46)
Fatigue	3.45 (2.86)	2.36 (2.58)	2.87 (2.75)	1.96 (2.32)	2.53 (2.78)	1.94 (2.43)	2.57 (2.67)	1.87 (2.39)	2.03 (2.61)	1.90 (2.31)	1.78 (2.20)	1.80 (2.23)	2.39 (2.66)	1.90 (2.34)
Nausea	1.03 (2.03)	0.56 (1.59)	0.74 (1.62)	0.28 (0.97)	0.64 (1.70)	0.22 (0.83)	0.55 (1.61)	0.41 (1.51)	0.40 (1.40)	0.24 (0.84)	0.51 (1.37)	0.40 (1.28)	0.57 (1.58)	0.32 (1.16)
Disturbed sleep	2.47 (3.41)	1.92 (3.00)	1.70 (2.54)	1.57 (2.53)	1.87 (2.84)	1.26 (2.26)	2.05 (2.68)	1.56 (2.41)	1.83 (2.53)	1.44 (2.34)	1.95 (2.48)	1.23 (2.10)	1.93 (2.68)	1.42 (2.34)
Distress/feeling upset	2.55 (3.12)	1.72 (2.25)	2.00 (2.70)	1.84 (2.24)	1.63 (2.54)	1.09 (2.12)	1.58 (2.27)	1.39 (2.32)	1.58 (2.40)	1.18 (2.13)	1.54 (2.22)	1.09 (1.83)	1.68 (2.46)	1.27 (2.14)
Shortness of breath	0.88 (1.88)	0.58 (1.34)	0.64 (1.60)	0.99 (2.11)	0.66 (1.76)	0.72 (1.77)	0.67 (1.69)	0.85 (1.78)	1.20 (2.30)	1.19 (2.12)	1.45 (2.43)	1.36 (1.98)	0.93 (2.02)	1.02 (1.94)
Difficulty remembering	1.39 (2.64)	0.64 (1.48)	1.07 (2.12)	0.62 (1.72)	1.17 (2.24)	0.67 (1.52)	0.98 (1.83)	0.78 (1.57)	0.86 (1.65)	0.88 (1.50)	1.01 (1.63)	1.40 (2.11)	1.03 (1.94)	0.89 (1.69)
Lack of appetite	0.83 (1.92)	1.00 (1.97)	0.80 (2.07)	0.38 (1.34)	0.34 (1.12)	0.36 (1.22)	0.47 (1.35)	0.52 (1.60)	0.53 (1.62)	0.54 (1.49)	0.66 (1.61)	0.55 (1.34)	0.55 (1.55)	0.51 (1.46)
Drowsiness	2.66 (2.62)	2.17 (2.74)	2.26 (2.71)	1.89 (2.51)	1.96 (2.55)	1.84 (2.31)	1.98 (2.49)	1.75 (2.46)	1.57 (2.26)	1.73 (2.20)	1.63 (2.26)	1.56 (2.09)	1.89 (2.46)	1.75 (2.31)
Dry mouth	0.55 (1.23)	0.92 (2.03)	0.59 (1.72)	0.59 (1.74)	0.65 (1.86)	0.68 (1.69)	1.08 (2.22)	0.76 (1.80)	1.14 (2.22)	1.10 (2.18)	1.66 (2.54)	1.40 (2.32)	1.02 (2.14)	0.95 (2.02)
Sadness	2.15 (2.70)	1.67 (2.88)	1.84 (2.89)	1.16 (1.86)	1.48 (2.44)	1.03 (2.04)	1.34 (2.21)	1.19 (2.19)	1.29 (2.32)	1.02 (1.94)	1.22 (2.16)	1.14 (2.17)	1.44 (2.39)	1.12 (2.10)
Vomiting	0.58 (1.81)	0.17 (0.70)	0.30 (1.16)	0.31 (1.11)	0.26 (1.26)	0.11 (0.63)	0.26 (1.23)	0.23 (1.09)	0.19 (1.03)	0.26 (1.04)	0.35 (1.18)	0.25 (1.11)	0.28 (1.22)	0.22 (1.00)
Numbness/tingling	0.57 (1.37)	0.50 (1.40)	0.71 (1.67)	0.59 (1.57)	0.92 (2.21)	0.74 (1.76)	0.85 (1.85)	0.82 (1.88)	1.01 (2.11)	0.80 (1.89)	1.11 (2.07)	0.85 (1.76)	0.91 (1.99)	0.77 (1.79)
Interference items														
General activity	2.17 (2.66)	1.44 (2.20)	1.75 (2.52)	1.65 (2.52)	1.94 (2.80)	1.65 (2.55)	1.83 (2.65)	1.54 (2.44)	1.95 (2.70)	1.49 (2.43)	1.78 (2.63)	1.39 (2.14)	1.88 (2.67)	1.52 (2.30)
Mood	2.69 (2.87)	2.08 (2.38)	2.30 (2.69)	1.73 (2.28)	1.96 (2.67)	1.77 (2.50)	1.56 (2.28)	1.25 (1.99)	1.38 (2.21)	1.15 (1.93)	1.34 (2.02)	1.20 (2.03)	1.71 (2.42)	1.38 (2.14)
Working	2.31 (2.92)	1.92 (2.68)	2.29 (3.00)	1.68 (2.38)	2.12 (2.96)	1.84 (2.70)	1.96 (2.75)	1.44 (2.43)	1.99 (2.78)	1.32 (2.30)	1.98 (2.74)	1.38 (2.20)	2.06 (2.83)	1.51 (2.41)
Relations with other people	2.20 (2.76)	1.89 (2.71)	1.89 (2.66)	1.41 (2.52)	1.61 (2.49)	1.46 (2.42)	1.30 (2.17)	1.00 (1.98)	1.38 (2.39)	0.96 (1.78)	1.04 (2.00)	1.02 (2.00)	1.45 (2.37)	1.15 (2.11)
Walking	0.45 (1.49)	0.36 (1.44)	0.34 (0.93)	0.67 (1.97)	0.77 (2.05)	0.61 (1.68)	1.05 (2.22)	0.86 (2.14)	1.51 (2.79)	1.02 (2.13)	1.79 (2.99)	1.32 (2.41)	1.11 (2.39)	0.91 (2.10)
Enjoyment of life	1.68 (2.50)	1.83 (2.71)	1.58 (2.56)	1.44 (2.42)	1.49 (2.52)	1.37 (2.32)	1.36 (2.22)	1.20 (2.25)	1.47 (2.65)	1.18 (2.16)	1.41 (2.31)	1.27 (2.38)	1.46 (2.45)	1.29 (2.30)

*W* women, *M* men

^a^0 (“not present”) to 10 (“as bad as you can imagine”) NRS scale

Univariable regression analysis showed a significant positive association between the presence of one or more comorbidities (*p* < 0.001) and PHQ- score and MDASI sum score (*p* < 0.001). Level of education was also associated with MDASI sum score (*p* < 0.001), while no association was found with age (*p* = 0.5). Further, because of the low response rate in youngest age group separate analyses were done without this age group yielding similar results.

Multivariable linear regressions (Table 5) showed positive significant associations between the MDASI sum score and depression on the PHQ (*p* < 0.001) and the presence of one or more comorbidities (*p* < 0.001). Participants with the highest education level had significantly lower MDASI sum score than respondents with education in elementary and/or primary school (*p* = 0.006) and second level (high school) (*p* = 0.003). Women had significantly higher MDASI sum score than men in univariable analyses (*p* = 0.001), but not in the multivariable regression model. The overall model fit was *R*^*2*^ = 0.45.

**Table 5:** Multiple linear regression on the MDASI sum score with age, sex, education, comorbidity and depression as explanatory variables (*N* = 2021)

	MDASI sum score^a^		
	Adjusted *R*^*2*^ = 0.45		
	B	95% CI	p
Age groups			0.446
18–29 years	0.397	−2.761, 3.555	0.805
30–39 years	−0.449	−2.985, 2.087	0.728
40–49 years	− 1.214	−3.318, 0.890	0.258
50–59 years	0.735	−1.244, 2.715	0.466
60–69 years	0.292	−1.583, 2.167	0.760
70–80 years (ref)	–		
Sex			0.109
Women	0.99	−0.222, 2.202	0.109
Men (ref)	–		
Education			0.002
Elementary and/or primary school	2.591	0.759, 4.423	0.006
Second level (high school)	2.029	0.695, 3.363	0.003
Third level (university or university college) (ref)	–		
Comorbidities			0.000
0 (ref)	–		
1–2	3.452	2.116, 4.789	0.000
≥3	10.693	8.627, 12.760	0.000
Depression			0.000
PHQ score	4.685	4.412, 4.958	0.000

^a^Demographic and disease-related variables that were significantly correlated with MDASI sum score in the univariable analyses were entered as covariates

Each interference item was used as the dependent variable in separate multivariable linear regression analyses (Table 6), with age, sex, education, comorbidity, PHQ score and MDASI sum score as independent variables. Comorbidities, PHQ score and MDASI sum score were significantly associated with both general activity and work as the dependent variables (*p* ≤ 0.001). Increased number of comorbidities and higher MDASI sum score were significantly associated with higher score on the interference item walking (*p* < 0.001). Further, the multivariable regression analyses showed that PHQ score and MDASI sum score were significantly associated (*p* < 0.001) with mood, relations and enjoyment of life as dependent variables.

**Table 6:** Multiple linear regression with the six interference items as the outcomes for all respondents included (*N* = 2021) ^a^

	General activity			Mood			Working			Relations			Walking			Enjoyment of life		
	Adjusted *R*^*2*^ = 0.460			Adjusted *R*^*2*^ = 0.584			Adjusted *R*^*2*^ = 0.516			Adjusted *R*^*2*=^ 0.543			Adjusted *R*^*2*^ = 0.291			Adjusted *R*^*2*^ = 0.589		
	B	95% CI	p	B	95% CI	P	B	95% CI	p	B	95% CI	p	B	95% CI	p	B	95% CI	p
Age groups																		
18–29 years	0.100	−0.339, 0.539	0.655	0.66	0.32, 0.995	0.000	0.04	−0.39, 0.48	0.848	0.43	0.08, 0.77	0.016	− 0.98	−1.42, − 0.54	0.000	− 0.25	− 0.60, 0.11	0.176
30–39 years	0.181	0.173, 0.534	0.316	0.58	0.32, 0.85	0.000	0.27	− 0.08, 0.62	0.126	0.35	0.08, 0.63	0.012	−0.77	−1.12, − 0.42	0.000	− 0.10	−0.39, 0.18	0.473
40–49 years	0.381	0.087, 0.674	0.011	0.54	0.32, 0.76	0.000	0.37	0.08, 0.65	0.013	0.36	0.13, 0.59	0.002	−0.53	−0.83, − 0.24	0.000	− 0.02	− 0.26, 0.22	0.857
50–59 years	0.233	− 0.044, 0.509	0.099	0.13	− 0.08, 0.34	0.213	0.07	−0.20, 0.34	0.610	0.07	−0.15, 0.28	0.546	−0.38	−0.66, − 0.10	0.007	−0.06	− 0.28, 0.17	0.628
60–69 years		−0.036, 0.489	0.091	0.07	−0.30, 0.27	0.502	0.04	−0.22, 0.296	0.771	0.14	−0.07, 0.35	0.187	−0.14	−0.41, 0.12	0.287	0.06	−0.16, 0.27	0.609
70–80 years (ref)	–			–			–			–			–			–		
Sex																		
Women	0.084	−0.085, 0.253	0.331	0.02	−0.11, 0.15	0.75	0.22	0.06, 0.39	0.009	0.02	−0.12, 0.16	0.775	0.06	−0.11, 0.23	0.470	−0.13	−0.27, 0.01	0.066
Men (ref)	–			–			–			–			–			–		
Education			0.536	–														
Second level, first stage	0.145	−0.111, 0.402	0.267	–			0.11	−0.14, 0.36	0.387	–			0.36	0.10, 0.61	0.007	0.08	−0.13, 0.29	0.446
Second level, second stage	0.026	−0.160, 0.212	0.783	–			0.10	−0.09, 0.28	0.31	–			0.05	−0.14, 0.23	0.611	−0.14	−0.29, 0.02	0.077
Third level (ref)	–			–			–			–			–			–		
Comorbidities																		
0	−0.672	−0.967, −0.377	0.000	−0.07	−0.30, 0.16	0.530	−0.48	− 0.77, − 0.19	0.001	0.26	0.02, 0.50	0.033	−0.81	−1.10, − 0.51	0.000	−0.17	− 0.41, 0.07	0.160
1–2	−0.384	− 0.660, − 0.107	0.007	−0.01	− 0.22, 0.21	0.960	− 0.32	−0.60, − 0.05	0.020	0.34	0.12, 0.56	0.003	−0.78	−1.06, − 0.51	0.000	− 0.18	−0.41, 0.04	0.107
≥ 3 (ref)	–									–			–			–		
Depression																		
PHQ score	0.175	0.127, 0.224	0.000	0.34	0.30, 0.37	0.000	0.26	0.21, 0.31	0.000	0.36	0.32, 0.40	0.000	−0.03	−0.08, 0.02	0.224	0.47	0.43, 0.51	0.000
Symptom burden																		
MDASI sum	0.075	0.068, 0.081	0.000	0.06	0.06, 0.67	0.000	0.08	0.07, 0.08	0.000	0.06	0.05, 0.06	0.000	0.06	0.05, 0.07	0.000	0.05	0.05, 0.06	0.000

^a^ Demographic and disease-related variables that were significantly correlated with MDASI sum score in the univariable analyses were entered as covariates

## Discussion

This study provides the first Norwegian reference values for the MDASI based on data from 2021 men and women aged 18–80 years collected in 2015. The most frequent symptoms overall were fatigue, drowsiness and pain. Fatigue, pain and disturbed sleep had the highest mean scores. The mean scores for fatigue were highest in the youngest age group (18–29 years). The presence of one or more comorbidities, increasing levels of depressive symptoms and lower level of education were significantly associated with a higher MDASI sum score. Comorbidity showed the strongest association; having three or more comorbidities increased the MDASI sum score with 10 points in average. Sex was not significantly associated with MDASI sum score when education, depression and comorbidities were controlled for in the regression model.

The Health Study of Nord-Trøndelag County (HUNT 3) found that the prevalence of chronic pain was 36% among women and 25% among men, and that the prevalence increased with age [43]. A random sample of participants were followed with annual measures over 4 years [44]. Here, pain intensity ranging from no pain to very mild, mild, moderate, severe and very severe pain was included to identify clinically important pain. A cut-off between mild and moderate pain may identify individuals with complex pain [45]. In our study, a cut off ≥1 was chosen to identify the presence of a symptom. By increasing the cut off to ≥3, the prevalence was about 34% for pain, which corresponds to the finding in the HUNT 3 study.

Previous studies have shown that women generally report a higher number of symptoms than men [3, 5, 46, 47]. A Norwegian population study [47] also found that women reported a higher number of symptoms than men, although the association between somatic symptoms and anxiety and depression was equally strong in men and women indicating that the difference in prevalence of these conditions between the sexes could not explain the difference in the reported number of somatic symptoms. Elnegaard et.al [2]. found no sex differences for almost 2/3 of the reported symptoms leading to contact with a general practitioner in their population study. In our study, more women (15%) than men (9%) reported depression on the PHQ-9. This might explain why sex was not associated with symptom sum score when controlling for depression.

Across the lifespan, depression is almost twice as common in women as in men. The prevalence of major depressive episode worldwide is approximately 5% [48]. However, major depressive disorder is different from feelings of sadness which also may lead to increased symptom score. The PHQ-9 is a tool that can be used to identify and assess depression, but it is important to also assess contextual factors like alternative psychiatric diagnoses, a medical illness, or the side-effects of medication [49]. We used the PHQ-9 as a measure of depressive symptoms, and not as a measure of depressive disorder. Symptom criteria for depression overlap symptoms of cancer and other comorbidities, e.g. fatigue, poor appetite and sleep problems [50]. In patients with increased symptom burden, exclusion of somatic symptom criteria in the PHQ-9 may reduce the likelihood of being false positive categorized as depressed [42]. In this study, the four somatic depression symptoms in the PHQ-9 were excluded to avoid overlap with the MDASI. We found a significant association between higher levels of depressive symptoms and higher MDASI sum score.

Comorbidities were significantly associated with an increased MDASI sum score in our study. A cross-sectional study from the USA [51] found that symptom scores on all domains were significantly worse in people with multiple sclerosis than in the general population, also after adjusting for age and sex. Similarly, a study found that patients with systemic lupus erythematosus had symptom scores that indicated poorer average health status compared with the general population [52]. A survey among patients with type 2 diabetes in primary care found that the study population reported more problems with physical functioning and pain compared to the general population [53]. This illustrates the importance of reference values when comparing differences in daily function for populations with a specific disease and the general population. It is important to adjust for comorbidities when comparing different populations in terms of level of symptom scores. This also applies to other variables that significantly affect the symptom level, like depression and education. The independent variables included in the multiple regression model explained 45% of the variance in MDASI sum score. By controlling for relevant associated factors, potential bias is likely to be reduced.

Comorbidity, depression and MDASI sum score were significantly associated with the interference items general activity and work. Depression and MDASI sum score were negatively associated with enjoyment, mood and relations to other people. Bruusgaard et.al [4]. found a strong linear association between the number of self-reported symptoms and decreased functional status in the Norwegian Ullensaker population study. Anxiety and depression were symptoms that had substantially higher explanatory power on functional status than other symptoms [4]. This in in agreement with the findings in our study, with depressive symptoms being associated with all interference items but walking. These findings indicate that interference is influenced by other variables than just symptoms. This does not only apply to the emotional domains like enjoyment and mood, but also to the more functional ones like work and general activity.

### Limitations

The randomly drawn sample was assumed to be representative of the general Norwegian population with respect to age, sex, and place of living. However, only 36% of the sample responded to the survey. Compared to collection of Norwegian reference values for the SF-36 in 1996 and 2002 this response rate was low [32]. The decline in response rates from 67% in 1996 to 36% in 2015 is in line with other postal surveys [3, 23, 54, 55]. Another Norwegian study found that health-related quality of life was relatively stable in two cross-sectional studies over an 8 year period despite the response rate being 68% in the first study and 35% in the second [56]. Surveys are used to describe large populations, and high response rates are valued to reduce the risk of bias. However, nonresponse bias is only indirectly related to nonresponse rates and there is little empirical support for the notion that low response rates are more prone to nonresponse bias than samples with higher response rates [57]. The fact that response rates in sample surveys in general have declined over the past decades is challenging for population studies [57]. Innovation in epidemiologic studies should involve development of recruitment techniques that optimize participation [58]. A large Danish population study from 2015 [2] used web-based questionnaires and had a response rate of 52%. In our study, the paper-based questionnaire was not available in an electronic version.

The fact that a large proportion of the respondents had university level education may be considered as a potential bias regarding the representativity of the sample. According to Statistics Norway [59] 32% of the Norwegian population had higher education in 2015, 41% had finished high school and 27% had finished elementary school, corresponding to 46, 37 and 17% respectively in our study. This should be considered when using the reference values in groups with low education.

When comparing the sample to the actual composition of the Norwegian population, 15% of the population was 67 years or above in 2015, while 27% of the responders were in the same age group [32]. About 21% of the Norwegian population was between 18 and 29 years, while only 5% of this age group participated in the survey. The opposite pattern was seen for the older population. Thus, it is highly likely that the high mean scores for symptoms in the youngest age group are not entirely representative for the general population of the same age. The relatively high symptom scores in the youngest age group compared to the older age groups may indicate an unhealthy bias in the youngest age group and a healthy bias among the older age groups. Taken together, these factors suggest that the reference values might be biased due to selection among the youngest participants. Regrettably, our data did not permit further analyses to illuminate this.

In accordance with other frequently used PROMs-questionnaires, the MDASI assesses the most common cancer-related symptoms. The MDASI has been translated into and validated in several languages [27, 60, 61]. However, the MDASI has not gone through a complete psychometric validation in a Norwegian cancer population. Following our study this may be a natural next step, as the symptoms of the MDASI and the fact that it specifically assesses the interference with daily living caused by these symptoms, makes it a highly relevant tool for patient-centered care and follow-up. Such a study should also include other questionnaires- such as the Quality of Life Questionnaire-Core 30 (QLQ-C30) [62] and the Brief Pain Inventory (BPI) [63], both which are validated and frequently used in Norway. However, given that the MDASI symptoms are common among cancer patients, and that the answering format is similar to other tools, we assume the Norwegian MDASI to have both high face validity and convergent validity, as is also shown in studies from other countries [60, 61, 64].

## Conclusions

This study provides the first Norwegian reference values for the MDASI. The presence of one or more comorbidities, increased levels of depressive symptoms and lower level of education were significantly associated with higher MDASI sum score. These covariates must be controlled for when using the reference values.

## Data Availability

The dataset used and analysed during the current study is available from the corresponding author on reasonable request.

## Abbreviations

BPI: Brief Pain Inventory
EORTC: European Organisation for Research and Treatment of Cancer
FQ: Fatigue Questionnaire
HUNT: Health Study of Nord-Trøndelag
MDASI: M.D. Anderson Symptom Inventory
PHQ-9: Patient Health Questionnaire 9
PROMs: Patient Reported Outcome Measures
PROs: Patient Reported Outcomes
REC: Regional Committee for Medical and Health Research Ethics
SCQ: Self-Administered Comorbidity Questionnaire
SD: Standard Deviation
SF-36: Short-Form Health Survey-36
QLQ-C30: Quality of Life Questionnaire-Core 30
QoL: Quality of Life
